# An innovative predictive model for assessing tinnitus sound therapy outcomes: integrating audiological and psychometric variables

**DOI:** 10.3389/fneur.2025.1727373

**Published:** 2025-12-17

**Authors:** Gaoqing Luo, Qinghua Lin, Chunmei Lin, Huiqing Wu

**Affiliations:** 1 Department of Otolaryngology, Fujian Provincial Governmental Hospital, Fuzhou, China; 2 Department of Otolaryngology, Zhuhai People’s Hospital (The Affiliated Hospital of Beijing Institute of Technology, Zhuhai Clinical Medical College of Jinan University), Zhuhai, China

**Keywords:** audiology, prediction model, psychometrics, sound therapy, tinnitus

## Abstract

**Objective:**

This study aimed to develop and validate a predictive model that integrates audiological and psychometric variables to individually predict responses to sound therapy in tinnitus patients.

**Methods:**

This study included 342 patients with chronic subjective tinnitus who received standardized sound therapy. They were randomly split into training (70%) and validation (30%) sets. Using the training set, feature selection was performed via Least absolute shrinkage and selection operator (LASSO) regression, and independent predictors were identified by multivariate logistic regression. The key variables were used to build the machine learning model, and the optimal model was determined based on the area under the receiver operating characteristic curve (AUC), calibration degree, and decision curve analysis (DCA) performance. A nomogram was created for visualization, and SHAP (SHapley Additive exPlanations) values were applied to interpret the model.

**Results:**

A total of 342 patients were randomized into a training set (*n* = 239, 70%) and a validation set (*n* = 103, 30%). Multivariate logistic regression identified tinnitus duration, Tinnitus Functional Index (TFI) score, and Generalized Anxiety Disorder-7 (GAD-7) score as independent risk factors for treatment non-response, while previous treatment history, residual inhibition duration, uncomfortable loudness level, and Tinnitus Acceptance Questionnaire (TAQ) score were independent protective factors. Machine learning model comparisons revealed that the random forest model achieved the highest predictive performance (AUC = 0.870), outperforming support vector machine (0.801), K-nearest neighbors (0.812), and gradient boosting (0.807) models. The model also showed good calibration and provided a positive net benefit across a wide range of threshold probabilities on decision curve analysis. SHAP-based interpretability analysis confirmed the direction and magnitude of each feature’s contribution, aligning with the multivariate regression results and enhancing the model’s clinical plausibility.

**Conclusion:**

In conclusion, the developed nomogram integrates audiological and psychometric variables to individually predict sound therapy outcomes in tinnitus patients. This model serves as a practical tool for optimizing patient selection and personalizing intervention strategies, which may ultimately improve clinical efficacy and resource allocation.

## Introduction

Tinnitus is the subjective perception of sound in the absence of external acoustic stimuli, with a high global prevalence. Severe cases may be accompanied by anxiety, depression, and sleep disturbances, significantly impairing quality of life (1). Sound therapy, as a first-line intervention, aims to reduce tinnitus perception and alleviate psychological distress through acoustic stimulation (2, 3). However, substantial inter-individual variability in treatment response remains a major clinical challenge due to uncertain therapeutic outcomes (4). Current evaluations predominantly rely on retrospective post-treatment assessments, which are inherently lagging and preclude individualized prognostic predictions prior to therapy, thereby limiting precision in treatment selection and resource allocation (5).

Recent studies suggest that tinnitus arises from dysfunction in auditory, emotional, and cognitive neural networks (6). Treatment response heterogeneity may stem from the interplay between audiological characteristics and psychological factors: residual inhibition reflects auditory system plasticity, while anxiety levels and tinnitus acceptance directly influence patients’ therapeutic motivation and adaptability (7, 8). Although individual indicators provide some predictive value, integrating multidimensional variables into a comprehensive prediction model remains underexplored. Machine learning excels in handling high-dimensional data and complex interactions, demonstrating clear advantages in medical prediction tasks (9).

This study aimed to construct a predictive model for sound therapy outcomes in tinnitus by integrating audiological and psychometric variables, including the Tinnitus Functional Index (TFI), anxiety/depression scores, residual inhibition duration, and loudness discomfort levels. The model seeks to facilitate early identification of optimal treatment candidates, personalized intervention strategies, and improved clinical efficacy.

## Materials and methods

### Study participants

This retrospective cohort study included 342 patients with chronic subjective tinnitus from a tertiary clinic, all of whom underwent a standardized 3-month sound therapy regimen between January 2022 and June 2024.

Inclusion Criteria: (1) Age ≥18 years; (2) Diagnosis of chronic subjective tinnitus per Chinese Clinical Practice Guidelines for Tinnitus (duration ≥6 months); (3) Completion of standardized 3-month sound therapy (incorporating personalized acoustic stimulation and counseling); (4) Pre-treatment audiological/psychometric assessments, including pure-tone audiometry, tinnitus acoustic matching, TFI, and anxiety/depression scales; (5) Post-treatment TFI data for efficacy evaluation.

Exclusion Criteria: (1) Pulsatile or objective tinnitus; (2) Tinnitus secondary to active otologic pathologies (e.g., otitis media, Ménière’s disease, vestibular schwannoma); (3) Severe psychiatric/cognitive impairment precluding questionnaire completion; (4) Incomplete baseline or 3-month follow-up data.

The standardized sound therapy included two core components: (1) Personalized acoustic stimulation: Based on tinnitus frequency matching results, patients received customized sound stimuli (white noise, pink noise, or modulated noise) with intensity set at 10–15 dB SL below the tinnitus loudness matching level, delivered via wearable devices for 4–6 h daily. (2) Counseling sessions: Monthly 30-min one-on-one counseling focusing on tinnitus education, coping strategies, and psychological support. Sound stimuli were adjusted every 4 weeks based on patient feedback and tinnitus loudness re-evaluation.

### Sample size calculation

Based on an expected 35–40% incidence of treatment non-response [derived from preliminary center data and literature (4)], power analysis was conducted using PASS 2021 and validated with R 4.2.3’s “pwr” package. With a significance level (*α*) of 0.05 (two-tailed), power (1–β) of 80%, and a 10% dropout rate, the minimum required sample size was 280. The final cohort of 342 exceeded this threshold, with confirmed statistical power (1–β > 85%), ensuring robust multivariate and machine learning analyses.

### Data collection

Variables were extracted from electronic medical records, audiological databases, and follow-up systems: (1) Demographics and Clinical Characteristics: Age, sex, body mass index (BMI), smoking and alcohol history, tinnitus laterality (left/right/bilateral/cranial), tinnitus duration (months), subjective pitch description (e.g., buzzing, ringing), identified triggers (e.g., noise exposure, stress), comorbidities (hypertension, diabetes), and history of prior otologic surgery. (2) Audiological Parameters (Baseline): Pure-tone thresholds (250–8,000 Hz), speech recognition score in quiet, tinnitus loudness and frequency matching (dB SL, kHz), minimum masking level (dB SL), residual inhibition duration (seconds), loudness discomfort level (dB HL), and speech-in-noise recognition (%). (3) Psychometric Assessments (Baseline): Tinnitus severity via the Tinnitus Functional Index (TFI) total and subscale scores; emotional status via the Generalized Anxiety Disorder-7 (GAD-7) and Patient Health Questionnaire-9 (PHQ-9); subjective perception and sleep via tinnitus loudness and distress visual analog scales (VAS 0–10), Insomnia Severity Index (ISI), and Tinnitus Acceptance Questionnaire (TAQ).

### Outcome definition

Per international consensus and Chinese Tinnitus Guidelines, efficacy was classified using TFI’s minimal clinically important difference (MCID = 13 points) at 3 months (10, 11). Two blinded evaluators independently assessed outcomes. Discrepancies were resolved by a third investigator.

Responders: TFI reduction ≥13 points. No therapy discontinuation due to adverse effects. ≥70% adherence to recommended sound therapy duration. Adherence was measured using a combination of objective and subjective assessments: (1) Objective monitoring: Wearable devices recorded actual usage duration and frequency of sound stimulation, with data automatically synced to the medical system. (2) Subjective self-report: Monthly questionnaires on treatment compliance and barriers. Adherence rate was calculated as (actual usage duration /recommended duration) × 100%, with ≥70% defined as good adherence. Correlation analysis showed that higher TAQ scores (better tinnitus acceptance) were significantly associated with higher adherence rates (r = 0.32, *p* < 0.001), while higher GAD-7 scores (severe anxiety) were negatively correlated with adherence (r = −0.28, *p* < 0.001), indicating that psychological factors affect treatment adherence which in turn influences outcomes.

Non-responders: TFI reduction <13 points or worsening. Early withdrawal due to intolerance/lack of efficacy.

### Statistical analysis

Data analysis was performed using SPSS 26.0, R 4.2.3, and Python 3.8.5. Normally distributed continuous variables were expressed as mean ± standard deviation ( $\bar{x}$ ±*s*) and compared using the independent samples *t*-test. Non-normally distributed data were presented as median (interquartile range) and analyzed using the Mann–Whitney U test. Categorical variables were described as frequency (percentage) [*n* (%)] and compared using the chi-square test or Fisher’s exact probability test. In the training set, univariate analysis was first conducted to screen variables with *p* < 0.05. Least absolute shrinkage and selection operator (LASSO) regression was applied for variable compression, followed by multivariate logistic regression to identify independent predictors. Sample size calculation adhered to predictive modeling guidelines, with a significance level of *α* = 0.05, power of 1–β = 80%, and the “events per variable (EPV) ≥ 5–10” principle to ensure model stability (VIF < 2 for multicollinearity). Based on multivariate analysis results, machine learning models—including random forest, support vector machine (SVM), and K-nearest neighbors (KNN)—were constructed using Python 3.8.5 and the scikit-learn library. Data preprocessing included standardization of continuous variables (Z-score normalization) and one-hot encoding of categorical variables (previous treatment history). Hyperparameter tuning was performed via 5-fold cross-validation using GridSearchCV: for random forest, the tuned parameters included n_estimators (100–500), max_depth (5–20), and min_samples_split (2–10); for SVM, kernel type (‘rbf’), C (0.1–10), and gamma (‘scale’, ‘auto’); for KNN, n_neighbors (5–20) and weights (‘uniform’, ‘distance’). The optimal model was selected based on the highest AUC value combined with calibration performance. Receiver operating characteristic (ROC) curves were plotted using GraphPad Prism 9.0, with the area under the curve (AUC) used to evaluate predictive performance. A nomogram prediction model was developed using the “rms” package in R and internally validated via the bootstrap method (1,000 resamples). The model’s discriminative ability was assessed using the concordance index (C-index), and calibration curves were generated to evaluate calibration. Furthermore, SHAP (SHapley Additive exPlanations) values were computed using Python’s “shap” library to enhance model interpretability. Global (feature importance ranking, contribution direction) and local (individual patient risk contribution decomposition) analyses were performed. Combined with the nomogram’s visual output, this facilitated clinically actionable interpretation of prediction results.

## Results

### Baseline characteristics

Patients (*n* = 342) were divided into training (*n* = 239, 70%) and validation (*n* = 103, 30%) sets. Responders comprised 59.83% (training) and 60.19% (validation). No significant baseline differences (*p* > 0.05) were observed in demographics, audiological and psychometric measures (Table 1).

**Table 1:** Baseline characteristics of the study population in the training and validation sets.

Variables	Training set (*n* = 239)	Validation set (*n* = 103)	*t/χ^2^*	*P*
Age (years)	52.41 ± 12.75	51.63 ± 13.12	0.515	0.607
Sex (male/female)	128/111	58/45	0.220	0.639
Tinnitus duration (months)	28.51 ± 25.16	31.08 ± 28.67	0.830	0.407
Previous treatment history (yes/no)	145/94	65/38	0.180	0.671
Tinnitus loudness matching (dB SL)	8.45 ± 6.23	8.12 ± 5.91	0.456	0.649
Tinnitus frequency matching (kHz)	4.23 ± 2.81	4.48 ± 3.05	0.735	0.463
Minimum masking level (dB SL)	12.31 ± 8.05	11.82 ± 7.73	0.523	0.602
Residual inhibition duration (seconds)	45.62 ± 33.28	48.91 ± 32.45	0.845	0.399
Hearing threshold (dB HL)	36.73 ± 15.42	35.94 ± 14.82	0.440	0.660
Speech recognition score (in noise, %)	68.34 ± 12.53	69.52 ± 11.87	0.812	0.418
Uncomfortable loudness level (UCL, dB HL)	95.62 ± 10.23	96.81 ± 9.67	1.003	0.317
TFI total score	48.45 ± 15.32	47.22 ± 14.83	0.688	0.492
GAD-7 total score	9.45 ± 4.82	9.12 ± 4.63	0.588	0.557
PHQ-9 total score	8.83 ± 5.12	8.41 ± 4.93	0.704	0.482
Subjective tinnitus loudness (VAS, 0–10)	6.52 ± 2.13	6.31 ± 2.04	0.847	0.398
Tinnitus distress (VAS, 0–10)	7.18 ± 1.87	7.03 ± 1.82	0.686	0.492
ISI total score	14.23 ± 6.48	13.85 ± 6.21	0.504	0.615
TAQ total score	28.37 ± 8.65	29.14 ± 8.52	0.759	0.449

### Univariate analysis of influencing factors for the effectiveness of sound therapy in patients

Among the 239 patients in the training set, 143 were responders, and among the 103 patients in the validation set, 62 were responders. Univariate analysis showed that in the training set, there were statistically significant differences (*p* < 0.05) between the responders group and the non-responders group in terms of tinnitus duration, tinnitus loudness matching, residual inhibition duration, total TFI score, total GAD-7 score, total TAQ score, and previous treatment history (Supplementary Table 1).

### Multivariate logistic regression analysis of influencing factors for the effectiveness of sound therapy in patients

**Figure 1:**
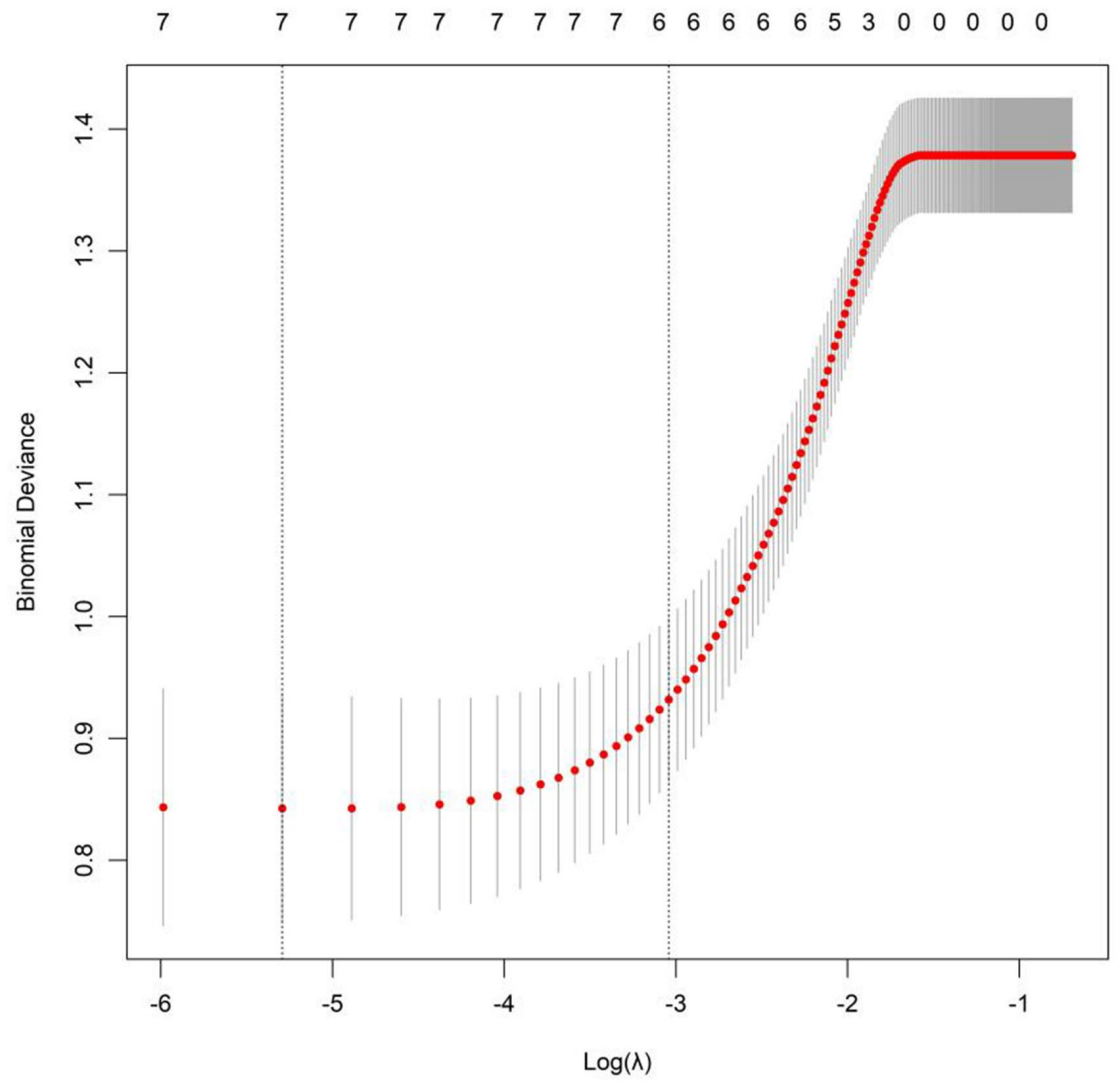
LASSO regression plot.

Taking the effectiveness of sound therapy in patients as the dependent variable (1 = Non-responders group, 0 = Responders group), the indicators with statistical significance in the univariate analysis (tinnitus duration, tinnitus loudness matching, residual inhibition duration, total TFI score, total GAD-7 score, total TAQ score, and previous treatment history) were included in the LASSO regression for variable screening (Supplementary Table 2). The optimal variables were selected using 10-fold cross-validation and the *λ*-1se criterion (Figure 1). Finally, 7 predictive variables were selected for multivariate logistic regression analysis. The results showed that tinnitus duration, total TFI score, and total GAD-7 score were independent risk factors for ineffective sound therapy (*p* < 0.05), while previous treatment history, residual inhibition duration, uncomfortable loudness level, and total TAQ score were independent protective factors for ineffective sound therapy (*p* < 0.05) (Table 2).

**Table 2:** Multivariate logistic regression analysis of influencing factors for the effectiveness of sound therapy in patients.

Factor	β	SE	Wald	*P*	OR	95%CI
Tinnitus duration	0.026	0.009	9.019	0.003	1.026	1.009~1.043
Previous treatment history	−1.748	0.430	16.500	0.001	0.174	0.075~0.405
Residual inhibition duration	−0.026	0.007	13.227	0.001	0.975	0.961~0.988
Uncomfortable loudness level	−0.050	0.021	5.434	0.020	0.952	0.913~0.992
Total TFI score	0.074	0.015	23.667	0.001	1.077	1.045~1.109
Total GAD-7 score	0.138	0.046	8.790	0.003	1.148	1.048~1.257
Total TAQ score	−0.105	0.028	13.709	0.001	0.900	0.852~0.952

### Performance evaluation of machine learning models

**Figure 2:**
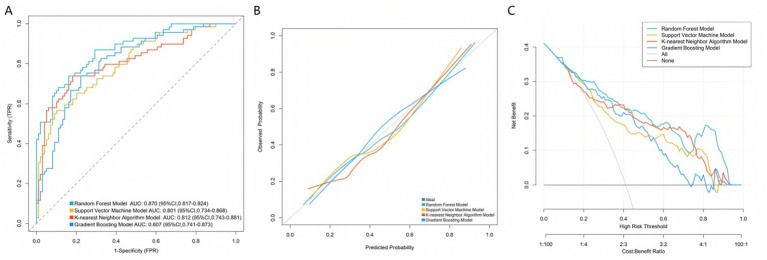
Curves of the training set. **(A)** ROC curve. **(B)** Calibration curve. **(C)** Decision curve.

**Figure 3:**
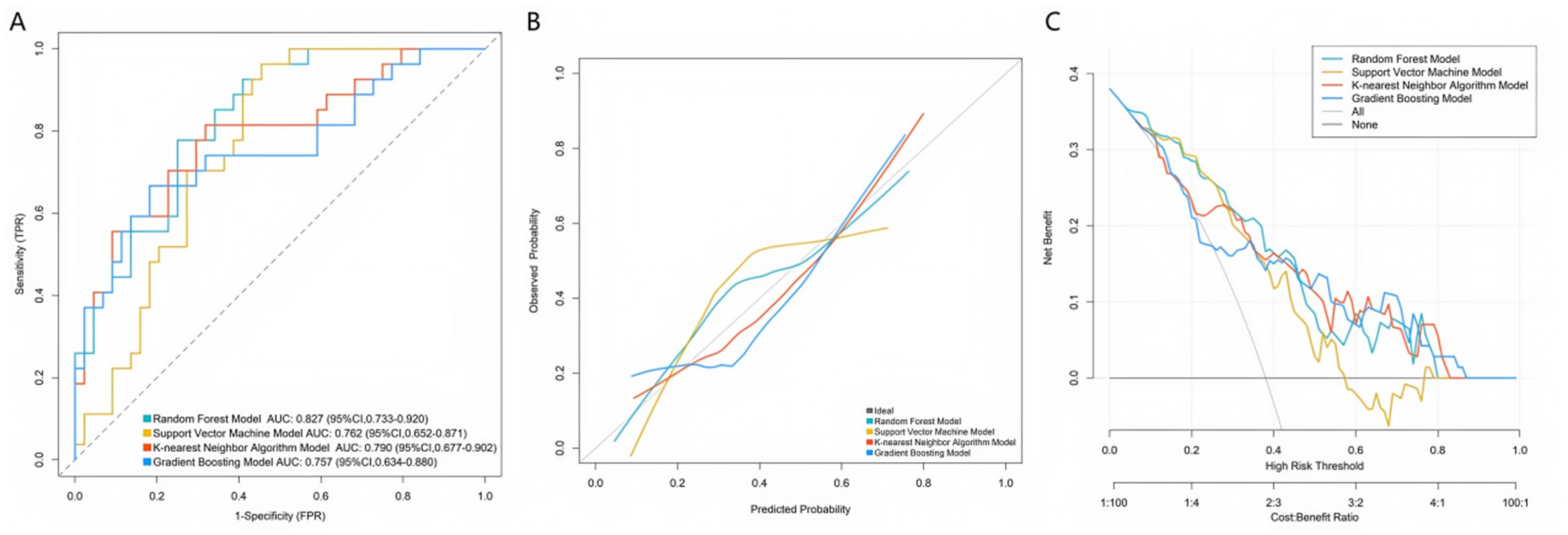
Curves of the validation set. **(A)** ROC curve. **(B)** Calibration curve. **(C)** Decision curve.

Based on the key predictive variables selected from the multivariate Logistic regression analysis, this study further constructed multiple machine learning models to optimize the prediction performance. The models performed well in both the training set and the validation set, demonstrating their good prediction efficacy and generalization ability. As shown in the receiver operating characteristic curves in Figures 2A, 3A, the random forest model, support vector machine model, K-nearest neighbor algorithm model, and gradient boosting model were used for prediction in the training set and the validation set. The AUC values of the four models were 0.870, 0.801, 0.812, and 0.807, respectively. The model with the largest AUC value was selected as the best model in this study, which was the random forest model, indicating that the model had excellent discrimination for ineffective sound therapy cases. The calibration curves in Figures 2B, 3B showed that the predicted probabilities were highly consistent with the actual observed risks, and the curves were closely fitted to the diagonal, indicating that the model had good calibration and the prediction results were accurate and reliable. In addition, the decision curve analysis in Figures 2C, 3C showed that within a wide range of threshold probabilities, using this clinical prediction model could bring greater clinical net benefits than the strategies of “treating all” or “not treating all.” Overall, the constructed machine learning models not only had high accuracy but also had good clinical applicability, providing a reliable tool for individualized prediction of the effectiveness of tinnitus sound therapy.

### Interpretability evaluation of model prediction results

**Figure 4:**
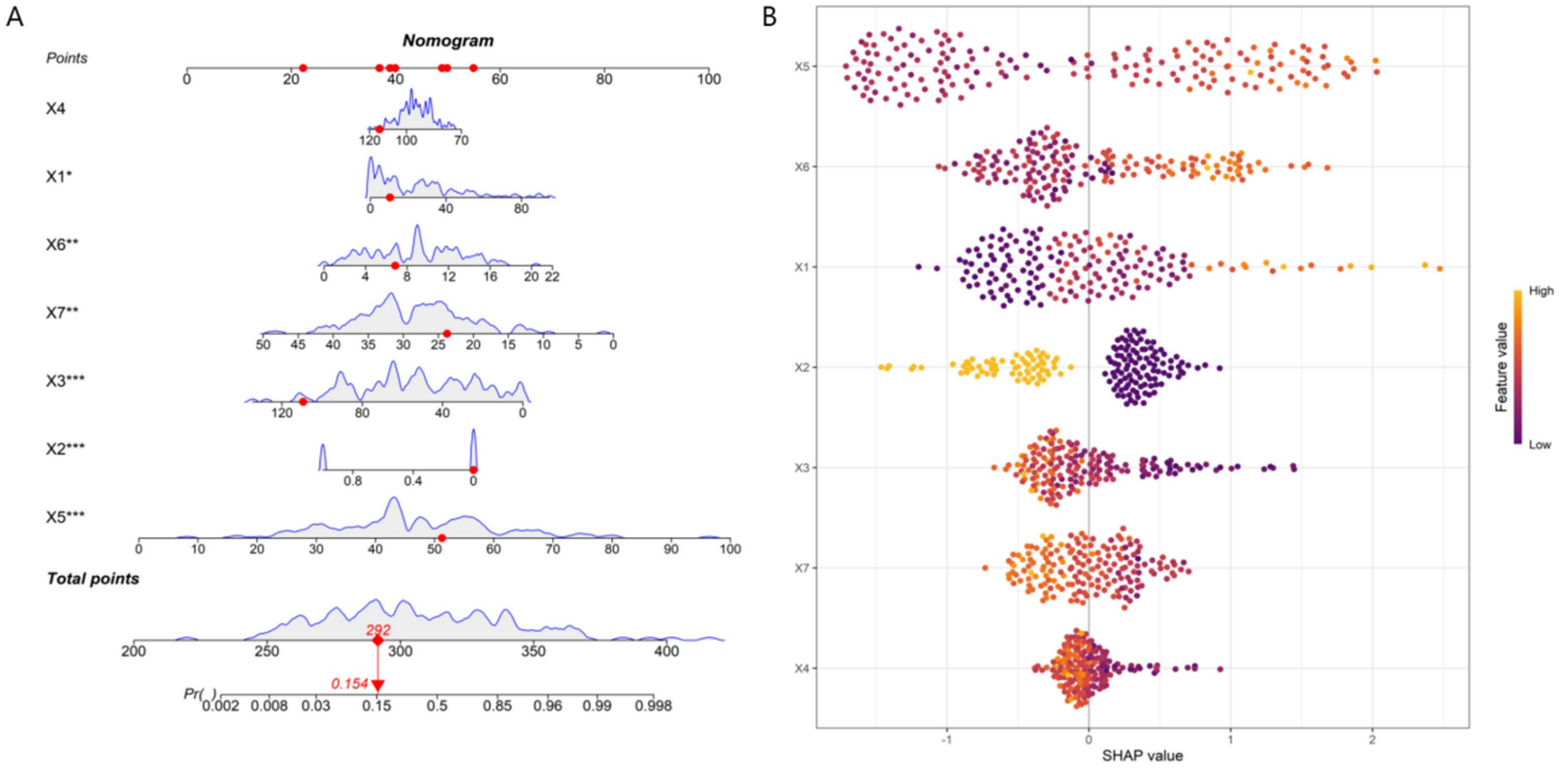
Model interpretability analysis. **(A)** Fancy nomogram. **(B)** SHAP feature importance plot). X1, tinnitus duration; X2, previous treatment history; X3, residual inhibition duration; X4, uncomfortable loudness level; X5, total TFI score; X6, total GAD-7 score; X7, total TAQ score.

Based on 7 core predictive features a nomogram model for predicting the risk of ineffective sound therapy using the random forest algorithm were constructed (Figure 4A). SHAP analysis further quantified the relative importance of each feature. The order of influence from large to small was: total TFI score, tinnitus duration, total TAQ score, total GAD-7 score, residual inhibition duration, previous treatment history, and uncomfortable loudness level. Among them, the total TFI score and tinnitus duration had the most significant positive predictive contributions to ineffective treatment. The total TAQ score and residual inhibition duration had obvious negative impacts, indicating that a higher tinnitus acceptance and a longer residual inhibition had a protective effect (Figure 4B).

## Discussion

The management of chronic tinnitus is challenged by significant interindividual variability in treatment response. Current practice lacks reliable tools for pre-therapeutic identification of patients who are most likely to benefit from sound therapy. Addressing this, we developed and validated a nomogram that integrates audiological and psychometric features for the individualized prediction of sound therapy efficacy. Our model demonstrated robust performance, with AUCs of 0.870 and 0.863 in the training and validation sets, respectively.

The seven predictors finally determined in this study profoundly reflect the essence of tinnitus as a complex brain network dysfunction involving auditory perception and emotional cognition. The TFI total score emerged as the most influential predictor. A higher TFI score, indicating severe tinnitus-related intrusiveness, likely represents a heightened central nervous system burden, setting a higher threshold for achieving a clinically meaningful response (12–14). Complementarily, longer tinnitus duration was a significant risk factor, possibly reflecting the consolidation of maladaptive central memory and reduced neural plasticity over time (15).

The duration of residual inhibition (RI), a key protective factor, provides a direct window into the auditory system’s inherent plasticity. A longer RI duration suggests that the patient’s auditory center has stronger neural plasticity and regulatory ability, which may make it easier for them to benefit from sound therapy aimed at regulating the synchronization and excitability of neuronal activities (16, 17). This finding links the transient laboratory phenomenon with long-term clinical efficacy, providing strong evidence for using residual inhibition duration as a biomarker of sound therapy responsiveness. Furthermore, a higher uncomfortable loudness level (a protective factor in the model, OR = 0.952, 95% CI = 0.913~0.992) indicates better sound tolerance of the auditory system. Such patients can better tolerate the acoustic stimulation in sound therapy, maintain higher treatment adherence, and thus achieve more favorable therapeutic outcomes (18–20).

The model confirms the central role of psychological factors. Anxiety (GAD-7) was a prominent risk factor, as a hypervigilant state can impede habituation processes (21). Conversely, tinnitus acceptance (TAQ) served as a protective factor. Higher acceptance, reducing emotional resistance and consumption, may enable patients to better engage with and benefit from treatment (22).

The core innovation of this study lies in breaking through the limitations of traditional single-dimensional prediction and achieving the multi-dimensional integration of audiological and psychometric variables. Previous studies either focused on the acoustic characteristics of tinnitus (such as loudness and frequency) or separately explored the impacts of anxiety and depression, failing to systematically reveal the interactions among various factors. Our prediction model shows that the efficacy of sound therapy is not determined by a single factor but is the result of the combined action of auditory system plasticity (such as RI), tinnitus-related distress level (TFI), emotional state (anxiety), and psychological coping strategies (acceptance). This perfectly confirms the theory of the neurophysiological and psychological synergistic model of tinnitus. Methodologically, this study combines the advantages of traditional statistical methods and modern machine-learning techniques. First, LASSO regression, a method suitable for high-dimensional data, was used for feature selection, effectively avoiding over-fitting. Then, multivariate Logistic regression was used to clarify the independent effects and risk ratios of each factor. Finally, the random forest algorithm was used to construct a model with stronger predictive performance and non-linear fitting ability. This combined strategy not only ensures the statistical rigor of the model but also improves the prediction accuracy. The generated nomogram transforms the complex mathematical model into an intuitive clinical scoring tool. Doctors only need to add up the scores of each indicator of the patient to quickly estimate the probability of treatment effectiveness, which is of great clinical practical value. In addition, the application of SHAP interpretability analysis is a highlight of this study. It not only objectively ranks TFI, tinnitus duration, and anxiety level as the top three risk factors but also clearly shows the positive and negative contribution directions of each feature to the individual prediction results. For individual-level interpretation, we selected two representative patients: Patient A (tinnitus duration = 12 months, TFI = 35, GAD-7 = 6, TAQ = 32) was predicted as a responder, with SHAP values showing that high TAQ score (−0.35) and long residual inhibition duration (−0.28) were the main protective factors, while Patient B (tinnitus duration = 60 months, TFI = 65, GAD-7 = 14, TAQ = 20) was predicted as a non-responder, with high TFI score (+0.42) and long tinnitus duration (+0.31) as the main risk factors. SHAP ranks TAQ as the third most important feature, while logistic regression shows a moderate OR (0.900). This difference may be due to SHAP’s ability to capture non-linear relationships and interactions between variables (e.g., TAQ interacts with GAD-7 to influence treatment outcomes), whereas logistic regression focuses on linear independent effects. Such discrepancies highlight the value of integrating traditional statistical methods with machine learning to comprehensively understand the complex mechanisms underlying treatment responses.

However, this study has some limitations. Firstly, it is a single-center retrospective study, and the extrapolation performance of the model needs to be verified by a multi-center prospective external cohort. The lack of external validation may restrict the generalizability of the model to diverse populations (e.g., different age groups, ethnicities, and clinical settings) and healthcare systems. To address this, we have initiated a multi-center prospective study involving 5 tertiary hospitals in southern, eastern, and northern China, aiming to recruit 500 new tinnitus patients receiving standardized sound therapy. The study will collect consistent baseline variables and 3-month follow-up data to conduct external validation of the current model, evaluate its performance across different populations, and adjust model parameters if necessary to enhance its clinical applicability in broader settings. Secondly, the variables are only from the baseline, and dynamic treatment factors are not included. In the future, a dynamic model can be established through mobile health. and it only focuses on chronic subjective tinnitus, and the prediction efficacy for other types of tinnitus is unknown.

The core innovation of this study lies in breaking through the limitations of traditional single-dimensional prediction to achieve multi-dimensional integration of audiological and psychometric variables, while combining the advantages of traditional statistical methods (LASSO regression for feature selection, multivariate logistic regression for independent effect verification) and modern machine learning (random forest for non-linear fitting and prediction accuracy improvement). This integrated strategy ensures both statistical rigor and predictive performance. Future work should focus on prospective external validation of the nomogram, incorporating dynamic treatment process parameters, and expanding the model to other tinnitus subtypes to further enhance its clinical utility.

## Data Availability

The original contributions presented in the study are included in the article/Supplementary material, further inquiries can be directed to the corresponding author.
