# Supplementary material for: An innovative predictive model for assessing tinnitus sound therapy outcomes: integrating audiological and psychometric variables
Source: Front Neurol. 2025 Dec 17;16:1727373. doi: 10.3389/fneur.2025.1727373 (PMC12753358; doi:10.3389/fneur.2025.1727373)
Supplement: Supplementary file 1 [file Table_1.DOCX]

**Supplemental Table 1.** Univariate analysis of influencing factors for the effectiveness of sound therapy in patients

| Variables | Responders group (n=143) | Non-responders group (n=96) | *t/χ²* | *P* |
| --- | --- | --- | --- | --- |
| Age (years) | 52.18±11.87 | 52.75±13.96 | 0.339 | 0.735 |
| Sex (male/female) | 78/65 | 50/46 | 0.140 | 0.708 |
| Tinnitus duration (months) | 18.27±16.35 | 35.82±27.64 | 6.159 | 0.001 |
| Previous treatment history (yes/no) | 68/75 | 19/77 | 8.373 | 0.004 |
| Tinnitus loudness matching (dB SL) | 8.23±5.87 | 8.76±6.72 | 0.645 | 0.519 |
| Tinnitus frequency matching (kHz) | 4.35±2.64 | 4.07±3.05 | 0.755 | 0.451 |
| Minimum masking level (dB SL) | 11.98±7.45 | 12.83±8.87 | 0.800 | 0.424 |
| Residual inhibition duration (seconds) | 58.45±28.37 | 40.83±28.46 | 4.701 | 0.001 |
| Hearing threshold (dB HL) | 36.25±14.78 | 37.42±16.35 | 0.575 | 0.566 |
| Speech recognition score (in noise, %) | 69.12±11.87 | 67.18±13.45 | 1.174 | 0.242 |
| Uncomfortable loudness level (UCL, dB HL) | 96.75±8.42 | 92.76±9.57 | 3.398 | 0.001 |
| TFI total score | 43.36±12.45 | 53.83±14.27 | 6.007 | 0.001 |
| GAD-7 total score | 7.92±3.64 | 10.35±4.82 | 4.434 | 0.001 |
| PHQ-9 total score | 8.45±4.78 | 9.36±5.57 | 1.349 | 0.179 |
| Subjective tinnitus loudness (VAS, 0–10) | 6.38±1.95 | 6.73±2.34 | 1.254 | 0.211 |
| Tinnitus distress (VAS, 0–10) | 6.95±1.72 | 7.52±2.05 | 0.897 | 0.371 |
| ISI total score | 13.87±5.94 | 14.78±7.15 | 1.069 | 0.286 |
| TAQ total score | 29.68±6.45 | 25.25±7.83 | 4.772 | 0.001 |

**Supplemental Table 2.** Variable assignment table

| Variable | Meaning | Assignment |
| --- | --- | --- |
| X1 | Tinnitus duration | Continuous variable |
| X2 | Previous treatment history | Non-continuous variable |
| X3 | Residual inhibition duration | Continuous variable |
| X4 | Uncomfortable loudness level | Continuous variable |
| X5 | Total TFI score | Continuous variable |
| X6 | Total GAD-7 score | Continuous variable |
| X7 | Total TAQ score | Continuous variable |
| Y | Effectiveness of treatment | 1=Non-responders group, 0=Responders group |
